# Improved *Helicobacter pylori* Eradication Rate of Tailored Triple Therapy by Adding *Lactobacillus delbrueckii* and *Streptococcus thermophilus* in Northeast Region of Thailand: A Prospective Randomized Controlled Clinical Trial

**DOI:** 10.1155/2015/518018

**Published:** 2015-06-08

**Authors:** Taweesak Tongtawee, Chavaboon Dechsukhum, Wilairat Leeanansaksiri, Soraya Kaewpitoon, Natthawut Kaewpitoon, Ryan A. Loyd, Likit Matrakool, Sukij Panpimanmas

**Affiliations:** ^1^Department of Surgery, Institute of Medicine, Suranaree University of Technology, Nakhon Ratchasima, Thailand; ^2^Suranaree University of Technology Hospital, Nakhon Ratchasima, Thailand; ^3^Pathological Unit, Institute of Medicine, Suranaree University of Technology, Nakhon Ratchasima, Thailand; ^4^School of Microbiology, Institute of Science, Suranaree University of Technology, Thailand; ^5^Family Medicine and Community Medicine, Suranaree University of Technology, Nakhon Ratchasima, Thailand; ^6^Parasite Research Unit, Suranaree University of Technology, Nakhon Ratchasima, Thailand; ^7^Faculty of Public Health, Vongchavalitkul University, Nakhon Ratchasima, Thailand

## Abstract

*Background and Aim*. To evaluate the effect of *Lactobacillus delbrueckii* subsp. *bulgaricus* and *Streptococcus thermophilus* to *Helicobacter pylori* eradication in different periods of therapeutic protocol. *Methods*. Infected patients were randomized to one-week tailored triple therapy (esomeprazole 20 mg bid, clarithromycin 500 mg bid/metronidazole 400 mg tid if clarithromycin resistant, and amoxicillin 1000 mg bid) with placebo (group 1, *n*=100); one week of pretreatment with probiotics (group 2, *n*=100); and one week of pretreatment with probiotic followed by one week of the same probiotics after treatment (group 3, *n*=100). *Result*. PP analysis involved 292 patients, 98 in group 1, 97 in group 2, and 97 in group 3. Successful eradication was observed in 229 patients; by PP analysis, the eradication rates were significantly higher (*P*<0.01, 95% CI; 0.71–0.97) in group 2 and group 3 than group 1. ITT analysis eradication rates were significantly higher in group 2 and group 3 than group 1 (*P*<0.01 95% CI; 0.72–0.87), and there is no significant difference between the three groups (*P*=0.32) in terms of adverse events. *Conclusion*. Adding probiotics before or before and after tailored treatment can improve *Helicobacter pylori* eradication rates. This trial is registered with Thai Clinical Trials Registry number: TCTR20141209001.

## 1. Introduction

*Helicobacter pylori* is a neutrophilic, gram-negative, and ureolytic organism that is able to colonize the human stomach and play a major role in the pathogeneses of chronic gastritis, peptic ulcers, gastric atrophy, and gastric malignancy [1].* Helicobacter pylori* eradication is current standard treatment and prevents chronic gastritis, peptic ulcer recurrence, and malignancy change [2]. However, the eradication rates with conventional standard triple therapy have become unacceptably low over the last, including Thailand [3]. The effectiveness of the most commonly used therapies has been increasingly compromised by the rapid emergence of antibiotic resistant strains of* Helicobacter pylori* and by poor compliance to treatment by patients [3, 4]. However, clarithromycin base triple therapy is still recommended in area where clarithromycin resistance is low, or when therapy is chosen based on pretreatment susceptibility testing. Resistance to amoxicillin has remained relatively stable, while resistance rates to clarithromycin have been steadily increasing. According to nationwide survey of* Helicobacter pylori* antibiotic resistance in Thailand [5], the result shows that antibiotic resistance was present in 50.3% including amoxicillin 5.2%, tetracycline 1.7%, clarithromycin 3.7%, metronidazole 36%, ciprofloxacin 7.7%, levofloxacin 7.2%, and multidrugs in 4.2% but unknown “*mutation patterns*” of drug resistant. In daily clinical practice [5]. A meta-analysis investigated whether a preparation of probiotic could improve* Helicobacter pylori* eradication rates and reduce side effects [6]. Many reports have suggested that probiotics can improve the* Helicobacter pylori* eradication rate by approximately 5–10%. However, some studies have reported that the administration of probiotics alone does not eradicate* Helicobacter pylori* and that probiotics supplementation to triple therapy does not increase the eradication rates [7, 8]. Clarithromycin resistant* Helicobacter pylori* strains represent the main cause of treatment failure. Prescribing an antibiotic for* Helicobacter pylori* eradication based on susceptibility testing is an approach that has been used clinically, allowing “*tailored treatment*” with marked improvements in treatment success. Indeed, high eradication rates have been obtained by tailoring the triple therapy to the resistance pattern of* Helicobacter pylori* [9].

This is the first study performed to evaluate true prevalence and mutation pattern of clarithromycin resistant in northeast region of Thailand and effect of administration probiotics containing yogurt (Suranaree brand) by Suranaree farm, Suranaree University of Technology, Nakhon Ratchasima, Thailand, which contains* Lactobacillus delbrueckii* subsp.* bulgaricus* and* Streptococcus thermophilus* to tailored triple therapy beneficially affects* Helicobacter pylori* eradication rates.

## 2. Patients and Methods

### 2.1. Patients

Three hundred patients diagnosed with* Helicobacter pylori* associated gastritis participated in this study from June 2014 to January 2015. The following exclusion criteria were applied: age below 18 or above 70 years, previous* Helicobacter pylori* eradication treatment before previous 2 months, gastric ulcer or duodenal ulcer, suspected or confirmed malignancy on endoscopy, significant medical illnesses and history of previous gastric surgery, pregnant or lactating women, and the use of antimicrobials or gastrointestinal medications like PPIs or bismuth compounds within the previous 2 months, refusing yogurt due to underlying disease such as DM and history of drug allergy in first line therapy. The study was performed in accordance with good clinical practice and the guidelines of the Declaration of Helsinki. All patients provided a written informed consent and the study protocol was approved by the Ethics Committee for Research Involving Human Subjects Suranaree University of Technology (EC-57-22).

### 2.2. Diagnosis of* Helicobacter pylori* Associated Gastritis

A diagnosis of* Helicobacter pylori* associated gastritis was made if* Helicobacter pylori* were seen on histopathological examination and the rapid urease test was positive. Finally, we prove bacterial infection by PCR method. A recent study from India [10] attempt to define “*gold standard*” of diagnostic tests to determine* Helicobacter pylori* infection status depends on the sensitivity and specificity. Both sensitivity and specificity of nested PCR have been reported to be 100%. In contrast, the sensitivity and specificity of serological, urea breath, fecal antigen, rapid urease tests, histopathology, PCR, and culture have been found to be 85% and 79%, 75%–100% and 77%–100%, 67%–100% and 61%–100%, 75%–100% and 84%–100%, 66%–100% and 94%–100%, 75%–100% and 84%–100%, and 55%-56% and 100%, respectively.

### 2.3. Biopsy Specimens

Biopsy was done according to the updated sydney classification system [11], which indicates sampling from 5 biopsy sites: one specimen each should be obtained from the lesser curvature of the corpus about 4 cm proximal to the angulus (1), from the lesser curvature (2), greater curvature of the antrum (3), both within 2 to 3 cm of the pylorus, from the middle portion of the greater curvature of the corpus, approximately 8 cm from the cardia (4), and from the incisura angularis (5).

### 2.4. Histological Analysis

Gastric tissue specimens for histological analysis were sent to pathologist. The hematoxylin and eosin stain and Giemsa stain were used for identification of* Helicobacter pylori*. The pathological analysis made by 5 pathologists of Bangkok Pathological Laboratory outside Suranaree University of Technology.

### 2.5. DNA Isolation

The DNA of* Helicobacter pylori* was extracted from frozen gastric tissue biopsy specimens was stored at a temperature of less than −20°C using the QIAamp DNA FFPE tissue kit (Qiagen, USA). The DNA extraction was performed according to manufacturer protocol. Briefly, ten tissue sections of 5 *μ*M thick were collected in 1.5 mL micro centrifuge tubes. The tissue specimens were placed in a microcentrifuge tube, and buffer ATL (180 *μ*L) and proteinase K (20 *μ*L) were added. The samples were mixed by vortexing and incubated at 56°C until the tissues were completely lysed. Buffer AL (200 *μ*L) was added to the samples, which were subsequently incubated at 70°C for 10 minutes. Next, 240 *μ*L of 100% ethanol was added to the samples, which were mixed by vortexing for 15 seconds. Each sample was placed in a QIAamp spin column and centrifuged at 8000 rpm for 1 minute. The columns were washed with AW1 buffer (500 *μ*L), and samples were centrifuged at 8000 rpm for 1 minute. AW2 buffer (500 *μ*L) was added to the column, and samples were centrifuged at 14 000 rpm for 3 minutes. Buffer AE (200 *μ*L) was added to each sample, and samples were incubated for 1 minute prior to centrifugation at 8000 rpm for 1 minute. Finally, the DNA was extracted from the tissue.

### 2.6. Detection of Point Mutations in the 23S rRNA Gene of* Helicobacter pylori* by Real-Time PCR

The mutation detection of 23S rRNA gene was performed by using the real-time PCR technique for template amplification. The hybridization fluorescent probe was utilized for PCR product detection. The real-time PCR procedure was accomplished by using LightCycler 480 instrument (Roche diagnostics, Neuilly sur Seine, France). The identifications of target PCR products were accomplished by melting curve analyses. The target PCR products were amplified by using the primers HPYS and HPYA as previously reported in the previous literature. 27PCR-RFLP can also detect the point mutation A2142C of the 23S rRNA gene associated with resistance of* Helicobacter pylori* to clarithromycin. The amplified products have a size of 267 bp. The hybridization probes include the one that is in the mutation sites of the 23S rRNA gene of* H. pylori*, the sensor probe. The sequence is 5-GGCAAGACGGAAAGACC-3 (nucleotides 2504 to 2520). This sensor probe is labeled by LC-red 640 at 5′ and phosphorylated at 3′. The anchor probe will be hybridized to the PCR product at the site 3 bp upstream to the sensor probe. The probe sequence is 5-TGTAGTGGAGGTGAAAATTCCTCCTACCC-3 (nucleotides 2473 to 2501, GenBank accession number U27270). The probe is labeled with fluorescein at 3′. 3 *μ*L DNA templates were subjected to PCR reaction in the final volume of 20 *μ*L. The reaction mixture consists of MgCl_2_ (25 mM), forward and reverse primers (20 M each), sensor and anchor probes (20 M each), and 2 *μ*L of FastStart DNA Master Hybridization Probes (Roche Diagnostics). PCR amplification comprised an initial denaturation cycle at 95°C for 10 min, followed by 50 amplification cycles (with a temperature transition rate of 20°C/s) consisting of 95°C for 0 s, annealing at 60°C for 10 s, and extension at 72°C for 17 s. After amplification a melting step was performed, consisting of 95°C for 0 s, cooling to 45°C for 30 s (with a temperature transition rate of 20°C/s), and finally a slow rise in the temperature to 85°C at a rate of 0.1°C/s with continuous acquisition of fluorescence decline. According to previous report using this real-time PCR protocol, this melting curve analysis can detect the possible three mutant genotypes along with the wild type according to different *T*_*m*_. The reported *T*_*m*_ of the wild type, A2121C, A2142G, and A2143G were 61.5, 58.0, 53, and 53.6°C, respectively.

### 2.7. Probiotic Containing Yogurt

The yogurt contains* Lactobacillus delbrueckii* subsp.* bulgaricus* and* Streptococcus thermophilus* inoculation rate 50 u/250 mL.* Lactobacillus delbrueckii* subsp.* bulgaricus* (>10^5^ CFU/serve) and* Streptococcus thermophilus* (>10^8^ CFU/serve) were obtained from Suranaree farm, Suranaree University of Technology, Nakhon Ratchasima, Thailand. At least 24 months from date of manufacture when stored according to recommendations. At +50°C (410°F) the shelf life was at least 6 weeks.

### 2.8. Symptoms and Safety Evaluation

The study was performed in accordance with good clinical practice and the guidelines of the Declaration of Helsinki. All patients provided a written informed consent and the study protocol was approved by the Ethics Committee for Research Involving Human Subjects Suranaree University of Technology (EC-57-22). All patients were asked to report associated symptoms at baseline and during follow-up, including diarrhea, metallic taste, nausea/vomiting, and rash. Any adverse event related to therapy was recorded and analyzed.

## 3. Study Design

This randomized, prospective single center was conducted at Endoscopic Unit, Suranaree University of Technology Hospital (SUTH), located in Suranaree University of Technology, Nakhon Ratchasima province in northeast region of Thailand. Three hundred of* Helicobacter pylori* associated gastritis patients were randomized into three groups using Random Number Generator by SPSS for Windows (version 16.0; SPSS, Chicago, IL, USA) were enrolled to one-week tailored triple therapy with placebo (esomeprazole 20 mg bid, clarithromycin 500 mg bid, or metronidazole 400 mg tid if clarithromycin resistant and amoxicillin 1000 mg bid; group 1, *n* = 100): one week of pretreatment with probiotics containing* Lactobacillus delbrueckii* subsp.* bulgaricus* and* Streptococcus thermophilus* (group 2, *n* = 100) and one week of pretreatment probiotic before tailored triple therapy then followed by one week of the same probiotics after treatment (group 3, *n* = 100). After completion of therapeutic protocol, rapid urease test and biopsy were performed by gastroscopy at least 4 weeks because we want to assess the gastric mucosa patterns after treatment with probiotic. Diagnosis of* Helicobacter pylori* associated gastritis was seen on histopathological examination and the rapid urease test was positive and finally proved by PCR. At the time of enrollment, a personal interview was conducted and a questionnaire was administrated. Patients were informed of the importance of full compliance, warned of adverse event, instructed to complete treatment, and provided with a contact number, in case they encountered problem. Compliance and adverse events for three groups were evaluated by direct questioning by a physician and pill counting. Compliance was considered to be satisfactory when drug intake or yogurt exceeded 90%.

## 4. Statistical Analysis

The eradication rates of* Helicobacter pylori* were determined by ITT and PP methods. All enrolled patients were included in the ITT analysis. However, for the PP analysis, patients that were wrong enrollment, lost to follow up, and taken less than 90% of the prescribed drugs or yogurt, or those that had dropped out due to adverse events were excluded. SPSS for Windows (version 16.0; SPSS, Chicago, IL, USA) was used for the statistical analysis. The eradication rate, baseline demographic data, and clarithromycin mutation pattern were compared by Student's *t*-tests. The eradication rate and 95% confidence intervals in each group were calculated for both the PP and ITT populations. All results were considered statistically significant when the *P* values were less than 0.05 (Figure 2).

## 5. Results

### 5.1. Patient Population

**Figure 1:**
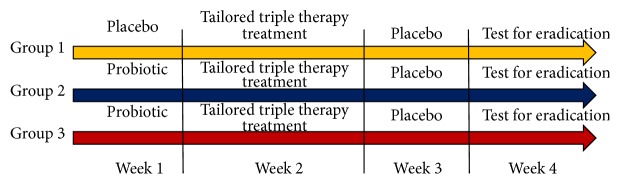
Flowchart of the study. Probiotic:* Lactobacillus delbrueckii* subsp.* bulgaricus* (>10^5^ CFU/mL),* Streptococcus thermophilus* (>10^8^ CFU/mL). Triple therapy: esomeprazole 20 mg bid, clarithromycin 500 mg bid, or metronidazole if clarithromycin resistant, and Amoxicillin 1000 mg bid.

**Figure 2:**
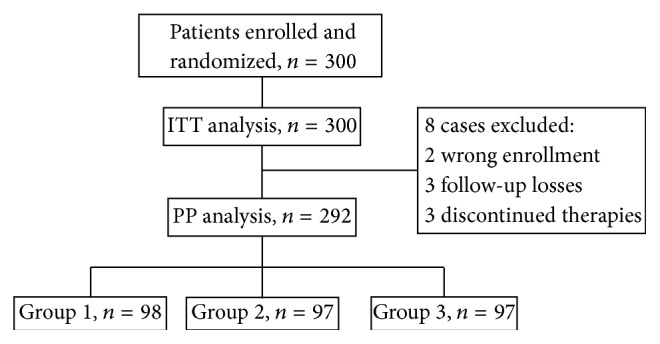
Flow diagram showing numbers of patients enrolled and missed for per protocol and intention-to-treat analyses. ITT: intention-to-treat; PP: per protocol. Group 1: triple therapy alone, group 2: pretreatment probiotic, and group 3: posttreatment probiotic.

**Figure 3:**
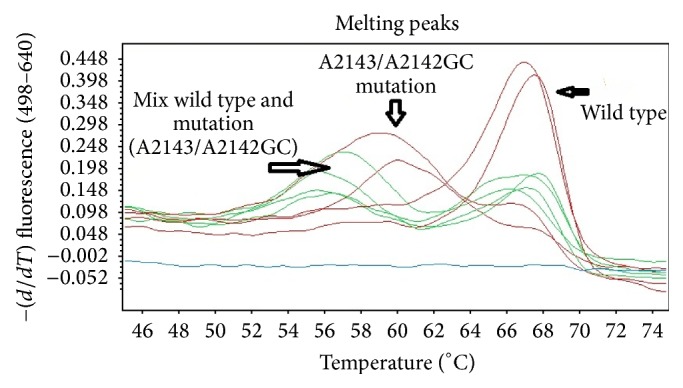
Pattern of clarithromycin resistance from real-time PCR.

Figure 1 shows a schematic diagram of this study. A total of 300* Helicobacter pylori* associated gastritis patients were enrolled into the study. Among these patients, 100 were assigned to the tailored triple therapy with placebo (group 1), 100 patients to one-week probiotic before the tailored triple therapy (group 2), and 100 patients to probiotic before and after tailored triple therapy (group 3). The demographic data of the 3 study groups are summarized in Table 1. Sex, the mean age of the patients, mean follow-up time, and pattern of clarithromycin resistance of the 3 groups were similar (Figure 3).

### 5.2. *Helicobacter pylori* Eradication

**Figure 4:**
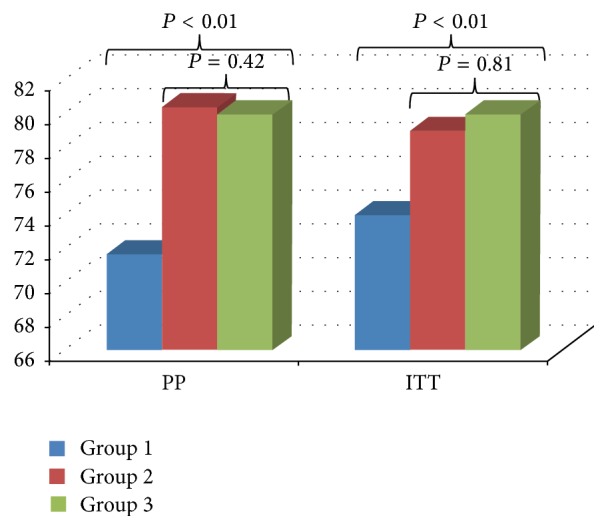
*Helicobacter pylori* eradication rate (ITT: intention-to-treat, PP: per protocol, Group 1: triple therapy alone, Group 2: pretreatment probiotic, and Group 3: pretreatment and posttreatment probiotic).

Four weeks after the completion of tailored triple therapy, by PP analysis* Helicobacter pylori* test on rapid urease test and biopsy was negative in 229 (78.42%) of the 292 patients. There were significant differences among 3 groups; however, the result showed that the eradication rates were significantly higher in group 2 (75/97, 77.3%) and group 3 (78/97, 80.4%) than group 1 (78/98, 74.5%) (*P* < 0.01, 95% CI 0.72–0.87) (Figure 4). ITT analysis showed that, significantly higher in group 2 (79/100, 79%), group 3 (80/100, 80%) and statistical significant (*P* < 0.01, 95% CI 0.71–0.97) than group 1.

### 5.3. Symptoms and Safety Assessment

The percentage of patients with adverse events was 11.82%, 9.78%, and 10.92% for the triple therapy with placebo, pretreatment probiotic, and pre- and posttreatment probiotic, respectively; there was no significant difference between 3 groups (*P* = 0.32) and only mild adverse event.

## 6. Discussion 

The effect of probiotics for* Helicobacter pylori* eradication was first discovered following a series of research studies in germ-free mice. The studies reported that* Helicobacter pylori* colonizes germ-free but not SPF mice and that* Lactobacillus* in the stomach of SPF mice inhibits colonization by* Helicobacter pylori* [12]. The beneficial effects of probiotics in the treatment of* Helicobacter pylori* associated gastritis have been supported by the majority of previous clinical trials in human subjects. These benefits included the increase in bacterial eradication rate and/or the decrease in the side effects of antibiotic treatment. Among these clinical trials, 12 studies were conducted to test the efficacy of the combination of probiotics and antibiotics for the treatment of* Helicobacter pylori* infection [13–24], whereas 16 studies investigated the effect of probiotics alone for the treatment of* Helicobacter pylori* gastritis [25–42]. Among those studies using probiotics as a complement during* Helicobacter pylori* eradication treatment, 6 in 12 trials showed increase in* Helicobacter pylori* eradication rate by adding probiotics. Six clinical trials also reported the decrease in adverse effects of antibiotic treatment. Overall 10 clinical trials in group showed some beneficial effects of probiotic complementary therapy. Among 16 clinical trials, which used probiotics alone for treatment of* Helicobacter pylori* associated gastritis, 3 studies reported improved eradication rate of the probiotic alternative treatment. However, 11 of 13 clinical trials which did not show increased eradication rate still showed the improved parameters of infection such as lower urea breath test, decreased bacteria colonization, or decreased gastric inflammation. Based on the previous clinical trials, 24 in 28 clinical trials indicated the benefit of using probiotics, alone or in combination with antibiotics for the treatment of* Helicobacter pylori* gastritis. However, with respect to the eradication rate, 9 in 28 clinical trials showed increased* Helicobacter pylori* eradication rate.

Tong et al. [6] conducted a meta-analysis of supplemental probiotics in eradication therapy including 14 randomized trials, and the eradication rates for triple therapy alone and eradication therapy plus probiotics were 74.8 and 83.6%, respectively. With combined treatment, the eradication rate increased, and adverse effects, such as diarrhea, decreased. However, the eradication rate varies by protocol. Sheu et al. [21] and Wang et al. [43] reported that pretreatment with* Lactobacillus* and* Bifidobacterium*-containing yogurt improved the efficacy of quadruple therapy after failed triple therapy. They also demonstrated a decreased bacterial load after pretreatment with yogurt. Therefore, we chose a protocol involving pretreatment with* Lactobacillus delbrueckii* subsp.* bulgaricus* and* Streptococcus thermophilus* containing yogurt in Group 2 and Group 3.

In our study, by using the real-time PCR hybridization probe method, the overall rate of mutation detection among the histologically has proven* Helicobacter pylori* infected cases were 76.71%. Among this positive group, the rate of cases which have wild type genotype of 23S rRNA gene is 23.28%. The proportion of the cases which have mutant strain only is 15.41% and those who have mixed wild type and mutant genotype were 61.30%. According to this result, the majority of histologically proven* Helicobacter pylori* infected cases have mutant genotypes, which confer the resistant property to clarithromycin. However, the clinical data indicated that most of the cases still respond well to the treatment protocol which included the combination of antibiotics accompanied with probiotic treatment. This observation indicated that, even in the cases who have resistant strain, this treatment protocol is still effective to eradicate the bacteria. The underlying reasons could be explained by these hypotheses. First, the clarithromycin resistant strains may still be sensitive to the two antibiotics included in the treatment protocol so the patient can be effectively treated. The second possibility is that the infection process can be influenced by adding the probiotics in the treatment protocol. The effect of each mechanism per se or the combination of both can also play a role. The possible reason that underlies the mixed genotypes is multiple infections of the same patient by two strains. The other is the occurring of mutation after one infection.

In conclusion, very high clarithromycin resistant in our area and mixed (wild type and mutant genotype) is the major mutant genotype. The further genotypic analyses have to pursue confirming these possible mechanisms. Pretreatment with probiotic or pretreatment with probiotic followed by posttreatment with the same probiotic can improve eradication rate of* Helicobacter pylori* eradication in both PP and ITT analysis; however, no significant between pre-treatment and post-treatment with the same probiotic in eradication rate and no significant difference between 3 groups about adverse event. Probiotic containing* Lactobacillus delbrueckii* subsp.* bulgaricus* and* Streptococcus thermophilus* containing yogurt (Suranaree band) are effective for additional treatment in tailored triple therapy.

## Figures and Tables

**Table 1:** Patient baseline demographics (PP, perprotocol analysis).

Patient baseline demographics	Tailored triple therapy with placebo (*n* = 98)	Probiotic before tailored triple therapy (*n* = 97)	Probiotic before and after tailored Triple therapy (*n* = 97)	*P* value
Male/female (*n*)	48/50	49/48	50/47	0.71

Mean age (years)	46.2	55.9	34.1	0.92

Mean follow-up time, (day)	33 ± 4	35 ± 2	34 ± 3	0.98

*Mutation pattern *				
(i) Wild type A2143/2142A (susceptible)	23	21	24	0.18
(ii) Mutation, A2143/2142CG (resistance)	15	18	12	0.14
(iii) Mixed wild type + mutation, A2143/A2142GC (Susceptible + resistance)	60	58	61	0.23
